# Independent and combined influences of physical activity, screen time, and sleep quality on adiposity indicators in Indian adolescents

**DOI:** 10.1186/s12889-021-12183-9

**Published:** 2021-11-15

**Authors:** Panchali Moitra, Jagmeet Madan, Preeti Verma

**Affiliations:** 1 Department of Food, Nutrition & Dietetics, Sir Vithaldas Thackersey College of Home Science (Autonomous), SNDT Women’s University, Santacruz West, Mumbai, Maharashtra 400049 India; 2 Department of Special Education, Department of Interdisciplinary Studies, SNDT Women’s University, Santacruz West, Mumbai, Maharashtra 400049 India

**Keywords:** Adolescent obesity, Clustering of unhealthy behaviors, Physical activity levels, Screen time, Sedentary behaviors, Sleep quality in adolescents, Obesity risk, Indian adolescents

## Abstract

**Background:**

Inadequate physical activity (PA), excess screen time (ST), and sub-optimal sleep quality tend to co-occur during adolescence. Yet, little is known about the associations of these behaviors as a cluster with adiposity indicators in Indian adolescents. This study aimed to evaluate the independent and combined influences of PA, ST, and sleep quality on body mass index (BMI) and waist to height ratio (WHtR) in 10–15 years old adolescents in Mumbai, India. A secondary aim was to explore if these influences vary between sexes.

**Methods:**

Cross-sectional study. Adolescents (*n* = 772, mean age 13.2 (1.4) years) reported frequency and duration of moderate to vigorous PA (MVPA) and time spent using screens on a previously validated instrument. Sleep quality was estimated using the Pittsburg Sleep Quality Index (PSQI). Weight, height, and waist circumference were measured. Mixed effect logistic regression analyses were performed to explore associations between adiposity indicators (BMI z scores > +1SD and WHtR > 0.5) and unhealthy behaviors (PA < 60 min/d, ST > 120 min/d and PSQI scores > 5), stratified by sex.

**Results:**

The combined prevalence of overweight and obesity was 38.3%. Overall, 62.0 and 85.0% reported MVPA< 60 min/d and ST > 120 min/d respectively. Girls reported higher ST (218.21 (69.01) min/d) as compared to boys (165.3 (101.22) min/d, *p* < 0.001). Clustering of low PA and excess ST was observed in 69.2% and of all three unhealthy behaviors in 18.8%. Among girls, MVPA < 60 min/d (OR = 1.78, 95% CI 1.54–1.92, p < 0.001) and PSQI scores > 5 (OR = 2.01, 95% CI 1.78–2.25, p < 0.001) predicted increased BMI. The odds of overweight/obesity were 2.10 times higher in boys reporting low PA and 4.13 times higher in those with low PA+ ST > 120 min/d. Clustering of all three unhealthy behaviors increased prevalence of obesity in both sexes.

**Conclusions:**

The results indicated a co-existence of multiple unhealthy lifestyle factors of obesity and that clustering of these behaviors can further aggravate obesity risk as compared to their independent effects. Integrated interventions that leverage the cumulative benefits of being active, less sedentary and sufficient sleep are warranted to facilitate greater improvements in obesity risk behaviors.

**Supplementary Information:**

The online version contains supplementary material available at 10.1186/s12889-021-12183-9.

## Introduction

The immediate and long-term health benefits of adequate physical activity (PA) levels, low recreational screen time (ST), and optimum sleep quality in adolescents are well documented [1–4]. Previous studies have shown significant associations of meeting the recommended moderate to vigorous physical activity (MVPA > 60 min/d) [5, 6], ST (≤ 2 h/d) [7–9], and sleep duration (9-11 h/d) [10, 11]) guidelines with a lower risk of obesity and its associated metabolic comorbidities during adolescence. However, despite these established guidelines, the PA levels are on a steady decline [7, 12], time spent in sedentary behaviors (SB) has increased [2, 13], sleep disturbances remain high [8, 11, 14, 15] and the prevalence of obesity continues to be on a progressive rise in adolescents across developed and developing countries [16, 17]. This necessitates a better understanding of the interactions between MVPA, SB, and sleep patterns in adolescents and the associations of the clustering of these behaviors with adiposity measures.

To date, studies have examined the independent effects of inadequate PA, and excess ST (such as time spent watching television, playing video games, and working or studying at the computers) on the body weight status of adolescents. Previous studies have consistently indicated an inverse association between PA and body weight status [18], a dose-response relation between reduced ST and improved weight-related outcomes [3], and the persistence of negative health effects of ST > 2 h/d independent of PA levels in adolescents [2]. The relationship between sleep patterns and adolescent obesity has also been investigated, though with mixed results. A higher prevalence of increased body mass index (BMI) and waist circumference (WC) was observed in adolescents with insufficient sleep [18–20], yet few studies reported that these associations were not statistically significant when adjusted for confounding variables [18, 21, 22]. The variations in results might be due to different age groups studied, instruments used to assess sleep patterns or the anthropometric measures evaluated as indicators of adiposity in participants. Similar to studies conducted outside India, the focus has largely been on the prevalence and correlates of reduced PA, high screen-based sedentary activities, and poor sleep quality in Indian adolescents [23–25]. Research regarding the clustering of these unhealthy behaviors in adolescents and the combined influences of not meeting the recommended PA, ST, and sleep-related guidelines on the adiposity measures remains limited.

The majority of the epidemiological studies have used BMI to characterize entire body fatness or general obesity [19, 26], despite its widely accepted limitation to accurately estimate body composition and regional body fatness. The shape-related indices such as waist circumferences may mitigate this limitation, however, are comparatively difficult to interpret due to varying cut-off thresholds between sex and ethnic groups [27]. In this context, determining waist to height ratio (WHtR) presents a simple indicator of central adiposity with robust discriminatory power and strong association with cardiometabolic risk factors in adolescents [27–30]. Given that both general and abdominal obesity are known to trigger an early onset of metabolic dysfunction during adolescence and increase the susceptibility to diabetes type 2 and cardiovascular diseases in adulthood [28, 31], exploring the associations between unhealthy lifestyle behaviors and different adiposity measures such as BMI and WHtR becomes imperative.

As the health risk behaviors tend to exist concurrently during adolescence [32, 33], there is a growing interest to examine the clustering patterns of movement (MVPA) and non-movement related activities (ST and sleep) over an entire 24 h period [13, 34]. Recently, several countries such as Canada, Australia, New Zealand, and South Africa have developed integrated 24 h movement guidelines to emphasize adherence to the right amounts of MVPA, recreational ST, and sleep per day for improved health outcomes [34–37]. However, the evidence supporting the beneficial effects of meeting these 24 h movement guidelines on obesity has been equivocal. For instance, a cross-sectional study showed no association between meeting all 24 h movement recommendations and adiposity indicators [38], another study reported a negative association between the number of guidelines met and adolescents’ body composition [39] and a multi-national study observed within and between-country variations in the recommended movement behaviors among participants [13].

Investigating the cluster of modifiable risk factors simultaneously in adolescents may help identify specific targets for culturally relevant behavior change interventions. We hypothesized that examining the clustering of low PA, excess ST, and poor sleep quality in adolescents in India and exploring whether the effect of one unhealthy behavior supersedes the effect of another on the risk of obesity can guide appropriate obesity prevention strategies. Therefore, we conducted this study to evaluate the independent and combined influences of lifestyle behaviors such as PA, ST, and sleep quality on the measures of adiposity – BMI and WHtR among 10–15 years old adolescents in Mumbai, India. The secondary objective was to determine whether these influences differed by sex. This information can help harness the synergistic benefits of improving PA, SB and sleep in adolescents in India and inform more targeted programs for prevention and management of obesity.

## Methods

### Study design, setting, and participants

This cross-sectional study was conducted among a sample of 10–15 years old adolescents (*n* = 772) attending three government-funded public schools and three unaided private schools in the city of Mumbai, India. A convenience sampling method informed the selection of the schools. In India, public schools are typically attended by children belonging to low and low middle socioeconomic class families and private schools usually cater to children from high middle to high socioeconomic class families. So, the selection of participants from private and public schools ensured representation across socioeconomic classes. At these six schools, a class each from grades 6 to 9 was selected at random as the participating class. As per the study design, all students (*n* = 988) attending these classes (*n* = 24, 4 classes each from 6 schools) were eligible to participate. Details of the protocol were explained, and information sheets were sent home to receive parental consent. Written informed assent was obtained from the adolescents who provided parental consent. The survey was administered at a central facility such as the library or school recreation center/auditorium in the presence of school teachers and a trained field research team. The study protocol was reviewed and approved by an independent research ethics committee, the Intersystem Biomedica Ethics Committee, Mumbai, India (ISBEC Protocol Ver 2 -February 2019).

### Sample size estimation

Based on the recent report released by the World Health Organization that showed the prevalence of insufficient physical activity to range from 66.8 to 80.0% in 11–17 years old Indian adolescents [40], we considered 25% as the prevalence of meeting the recommended physical activity levels. Using a 95% confidence level and a 5% margin of error, we estimated the sample size as 289. To derive proportional representation from private and public schools and after considering a non-response rate of 20%, the final sample size was estimated as 695.

### Measures

The self-reported survey included questions related to adolescents’ demographic characteristics (sex, date of birth, class of study, religion, and living arrangement), MVPA, recreational screen-based SB, and sleep quality. The parents provided information regarding the mother’s highest education and present occupation and the family’s monthly income while completing the parental consent forms. A separate section for the researcher recorded anthropometry measurements were also included.

#### Physical Activity levels and sedentary behaviors

Adolescents were asked to recall the frequency and duration of engaging in each of the listed MVPA and screen-based sedentary activities (SA) in the past week. A detailed description of the development and validation of these items included in this study is provided elsewhere [41]. Activities with MET values ≥3 such as playing football/ basketball, swimming, running, doing household chores, and more) were classified as MVPA and those with 1.5 MET values or lower (such as studying/ playing on a computer, watching television, and more) as sedentary, based on the Compendium of Physical Activities 2011. The weekly frequency responses ranged from ‘*never*’ to ‘*every day*’, scored 0 to 7 and the duration of engaging in each activity was reported in minutes/d.

The psychometric properties of the items assessing PA and SA were tested using several measures of validity and reproducibility. Item content validity indices for clarity and relevance were found to be satisfactory (> 0.80), the internal consistency (Cronbach’s alpha values for PA and SA were 0.881 and 0.920 respectively) was acceptable and the intraclass correlation coefficient estimates ranged from 0.954–0.968, indicating good to excellent reliability of the items included in the questionnaire. The total time spent in MVPA/d was calculated by multiplying the frequency of engaging in each activity with the time spent/d and then dividing the value by 7 (the number of days in a week). The time spent on each of the MVPA items was aggregated to estimate the total MVPA minutes/d/. Similar calculations were done to estimate the total time spent on ST/ d. Similar to previous studies [6, 42], MVPA ≥60 min/d and ST ≤ 2 h/d were considered as measures of adequate PA and ST in adolescents.

#### Sleep duration and quality

A widely used instrument, Pittsburg Sleep Quality Index (PSQI), assessed the adolescents’ sleep duration and quality during the previous month [43]. The questionnaire measures 7 sleep-related components- subjective sleep quality, sleep latency, sleep duration, habitual sleep efficiency, sleep disturbances, and daytime dysfunction, each scored 0–3. The global PSQI scores > 5 are considered indicative of poor sleep quality. The PSQI has shown excellent validity and acceptable reproducibility in adolescents in previous studies [44, 45].

#### Anthropometry measurements

Weight, height, and waist circumference (WC) were measured using standard procedures and calibrated equipment by trained field investigators. All field investigators had received systematic training for collecting and recording anthropometric measurements before data collection. Weight was measured standing erect and barefoot on an electronic weighing scale (Tanita, Japan) to the nearest 0.1 kg, height was taken using a portable stadiometer (Seca 213, Germany) to the nearest 0.1 cm and the WC was measured to the nearest 0.1 cm using a non-elastic measuring tape (Seca 203, Germany). The participants were asked to stand erect and WC was measured at the midpoint between the lowest part of the last rib and the iliac crest (top part of the hip bone) along the midline of the armpit*.* Each measurement was repeated twice and the average of these consecutive readings was used to calculate BMI using the formula- weight in kilogram divided by height in meter squared and WHtR by dividing WC in centimeters by height. The presence of overweight and obesity were determined by calculating BMI for age z scores using Anthro Plus software and then comparing with age-specific and sex-specific BMI z score cut-offs provided by the World Health Organization [46]. The BMI z scores > + 1 standard deviation (SD) and WHtR > 0.5 were defined as measures of adiposity. We used WHtR as an indicator of central obesity as it is considered a relatively easy and more robust predictor of cardiometabolic risk as compared to waist circumference, waist to hip ratio, or BMI [31, 47]. The age and sex-specific WC percentiles between 75th–90th and > 90th percentile were classified as centrally overweight and central obesity respectively [47, 48].

### Statistical analysis

Out of 988, a total of 874 (88.5% of those who were contacted) students provided written parental consent and comprised the study sample. After excluding students (*n* = 24) who were not present in school on the survey day and those (*n* = 78), who had provided > 30% incomplete information in the survey, the analyses were performed on the final sample (*n* = 772, 78.1% response rate) of adolescents, attending private (*n* = 382) and public schools (*n* = 390). Data were analyzed using IBM SPSS for Windows version 24.0 (IBM Corporation, Armonk, NY, USA). Descriptive characteristics were calculated as mean and SD or percentages for the entire sample and also compared between adolescent boys and girls using chi-square tests for categorical and t-tests for continuous variables respectively.

To investigate the independent and combined influence of unhealthy behaviors on adolescents’ adiposity measures, we categorized participants into four clusters- those who reported (1) low PA and excess ST, (2) low PA and poor sleep, (3) excess ST and poor sleep and (4) combination of all three unhealthy behaviors such as low PA, excess ST, and poor sleep. The cutoff points for low PA (engagement in MVPA < 60 min/ day), excess ST (time spent using screens > 2 h/d), and poor sleep (global PSQI score > 5) were operationalized based on the recent WHO 2020 report recommendations [42, 49] and the PSQI scoring methods [25, 50]. Mixed effect logistic regression analyses stratified by sex were performed to determine the association of predictor variables - PA (< 60 min/d), ST (> 2 h/d), sleep (global PSQI score > 5), and different combinations of unhealthy PA, ST and sleep behaviors with the dichotomized dependent variables- BMI z scores > +1SD and WHtR > 0.5 in separate models. Similar to previous studies, the demographic factors - age, type of school attended, mother’s highest educational qualification, and family’s monthly income were entered as covariates in the adjusted models. The adjusted odds ratio with their corresponding 95% confidence intervals (95% CI) were reported, considering a threshold of *p*-value ≤ 0.05 as a measure of statistical significance.

## Results

The mean age of the adolescents was 13.2 (1.4) years. Of 772 adolescents, 49.0% were girls, 50.5% attended public schools and 66.7% reported their living arrangement as nuclear families. The prevalence of overweight, and obesity were 27.8, and 10.5% respectively and 42.2% had WHtR > 0.5. The age-specific WC percentile data showed that 32.5% were centrally overweight (75th – 90th percentile) and 9.5% had central obesity (>95th percentile). Girls reported a higher prevalence of central obesity as compared to boys (Table 1).

**Table 1:** Demographic characteristics of 10–15 years old adolescents (*n* = 772) in the study

Characteristics	Overall (***n*** = 772)	Girls (***n*** = 378)	Boys (***n*** = 394)
**Age categories**			
10–12 years	396 (51.3)	204 (54.0)	192 (48.7)
13–15 years	376 (48.7)	174 (46.0)	202 (51.3)
**Type of school attended**			
Private School	382 (49.5)	192 (50.8)	190 (48.2)
Public School	390 (50.5)	186 (49.2)	204 (51.8)
**Grade in which studying**			
Sixth	190 (24.6)	97 (25.7)	93 (23.6)
Seventh	206 (26.7)	101 (26.7)	105 (26.6)
Eighth	209 (27.1)	98 (25.9)	111 (28.2)
Ninth	167 (21.6)	92 (24.3)	85 (21.6)
**Living Arrangement**			
Nuclear	515 (66.7)	263 (69.6)	252(64.0)
Joint	189 (24.5)	86 (22.8)	103(26.1)
Extended	68 (8.8)	29 (7.7)	39 (9.9)
**Mother’s highest education**			
Doctorate	72 (9.3)	43 (11.1)	29 (7.4)
Postgraduate	254 (32.9)	124 (32.8)	120 (30.5)
Graduate	279 (36.1)	158 (41.8)	121 (30.7)
12th/10th/8th Pass	167 (21.6)	53 (14.0)	124 (31.5)
**Mother’s present occupation**			
Service	156 (20.2)	75 (19.8)	81 (20.6)
Business	129 (16.7)	63 (16.7)	66 (16.8)
Doctor/Engineer/Lawyer	65 (8.4)	34 (9.0)	31 (7.9)
Self Employed	71 (9.2)	42 (11.1)	29 (7.4)
Unemployed/ Housewife	351 (45.5)	164 (43.4)	187 (30.5)
**Monthly family income in INR**			
< 30,000	128 (16.6)	52 (13.8)	76 (19.3)
< 50,000	185 (24.0)	83 (22.0)	102 (25.9)
50,000- 1,00,000	270 (35.0)	143 (37.8)	127 (32.2)
> 1,00,000	189 (24.5)	100 (26.5)	89 (17.2)
**Religion**			
Hindu	452 (58.5)	231 (61.1)	221 (56.1)
Muslim	196 (25.4)	86 (22.8)	110 (27.9)
Christian	44 (5.7)	26 (6.9)	18 (4.6)
Others (Jain, Sikh, Parsi, Buddhist)	80 (10.4)	35 (9.3)	45 (11.4)
**Bodyweight status**			
Underweight	98 (12.7)	55 (14.6)	43 (10.9)
Normal weight	378 (49.0)	187 (49.5)	191 (48.5)
Overweight	215 (27.8)	95 (25.1)	120 (30.5)
Obese	81 (10.5)	41 (10.8)	40 (10.2)
**Central Obesity**			
*Waist circumference* ^a^			
< 75th percentile	448 (58.0)	204 (54.0)	244 (61.9)
75-90th percentile	251 (32.5)	133 (35.2)	118 (29.9)
90th percentile	73 (9.5)	41 (10.8)	32 (8.1)
*WHtR > 0.5*	326 (42.2)	174 (46.0)	152 (38.6)

Data is presented as number (percentage) ^a^ age and sex-specific waist circumference percentiles

*Abbreviations*: *INR* Indian Rupee. *WHtR* Waist to Height Ratio

### Physical activity and sedentary behaviors

Among participants, the total time spent in MVPA was 68.33 (29.2) minutes/d, and the mean ST was reported as 172.41(88.32) minutes/d. Overall, 62.1% reported PA < 60 min/d and 85% engaged in ST > 2 h/d. Significant differences were observed in the mean frequency of performing activities such as football/basketball, swimming/bicycling, playing cricket/badminton, household chores between adolescent boys and girls but not in the total time spent in MVPA/ d. Also, girls reported spending significantly higher time in ST (218.21 (69.01) minutes/d) as compared to boys (165.34 (101.22) minutes/d, *p* ≤  0.001).

### Sleep quality

Analyses indicated that only 53.3% were sleeping > 7 h/d, 34.6% had < 75% habitual sleep efficiency (calculated using hours slept divided by hours spent in bed), almost three-fourths (74.6%) had experienced some sleep disturbances in the past month and 36.9% had PSQI scores > 5 (See Additional Table 1). Among this sample, 5.3% had trouble staying awake or engaging in social activity at least once or twice a week and 5.8% were experiencing ‘somewhat of a problem’ in keeping up the enthusiasm to get things done during the daytime.

The mean subjective sleep quality score was 1.62 (1.19), indicating ‘fairly good’ to ‘fairly bad’ sleep quality, and the mean sleep latency score was 1.29 (1.09), suggesting that adolescents usually took 15–30 min to fall asleep after going to bed. Girls had significantly higher mean global PSQI scores (7.12 (1.76)) as compared to boys (6.78 (2.02), *p* = 0.013) (Table 2).

**Table 2:** Physical activity levels, screen time and sleep quality of 10–15 years old adolescent boys and girls in the study

Variables	Overall (***n*** = 772)	Girls (***n*** = 378)	Boys (***n*** = 394)	***P*** value
**Moderate to Vigorous Physical Activity (MVPA)**	**Mean (SD)**	**Mean (SD)**	**Mean (SD)**	
Frequency of performing activities (d/week) ^a^				
a. Football/basketball/volleyball	2.15 (1.32)	1.09 (1.67)	3.36 (2.01)	< 0.001 ^**^
b. Running/jogging/ playing tag	2.20 (2.11)	2.18 (1.98)	2.25 (1.43)	0.572
c. Swimming/ bicycling	3.13 (2.01)	2.91 (1.08)	3.21 (1.69)	0.003 ^*^
d. Dancing/aerobics/martial arts	1.21 (1.06)	1.24 (1.01)	1.19 (1.06)	0.503
e. Cricket/badminton/ tennis	2.07 (1.22)	1.86 (1.22)	2.91 (1.20)	< 0.001^**^
f. Household chores (sweeping/ washing clothes)	1.31(1.04)	1.78 (1.03)	0.70 (1.01)	< 0.001^**^
Total time spent in MVPA (minutes/d)	68.33 (29.2)	70.33 (26.90)	66.21 (24.22)	0.091
MVPA < 60 min/d **(n%)**	479 (62.0)	242 (64.0)	237 (60.2)	0.253
**Screen-based Sedentary Behaviors (ST)**	**Mean (SD)**	**Mean (SD)**	**Mean (SD)**	
Frequency of performing activities (d/week)				
a. Studying/ playing on desktop/laptop	5.27 (1.11)	5.15 (2.21)	5.34 (1.78)	0.188
b. Watching television/ streaming platforms	6.89 (0.09)	6.77 (1.88)	6.92 (1.01)	0.165
c. Using social media	4.56 (1.87)	4.68 (2.02)	4.41 (1.66)	0.042^*^
d. Reading e-books/listening to music	3.13 (2.66)	3.56 (1.11)	2.98 (1.08)	< 0.001^**^
e. Watching videos/ movies	3.44 (1.91)	3.02 (1.56)	3.86 (2.02)	< 0.001^**^
f. Playing handheld video games	2.68 (1.05)	2.19 (1.03)	3.22 (1.11)	< 0.001^**^
Total time spent in ST (minutes/d)	172.41 (88.32)	218.21 (69.01)	165.34 (101.22)	< 0.001 ^**^
Screen time > 120 min/d **(n%)**	656 (85.0)	336 (88.9)	320 (81.2)	0.003^*^
**Sleep Quality**	**Mean (SD)**	**Mean (SD)**	**Mean (SD)**	
Component 1: Subjective sleep quality	1.62 (1.19)	1.59 (1.03)	1.64 (1.01)	0.496
Component 2: Sleep Latency	1.29 (1.09)	1.26 (1.01)	1.30 (1.04)	0.786
Component 3: Sleep Duration	1.51 (1.22)	1.68 (1.11)	1.42 (0.95)	0.005 ^*^
Component 4: Sleep Efficiency	1.01 (0.77)	1.01 (0.99)	1.02 (1.01)	0.712
Component 5: Sleep Disturbances	1.22 (0.99)	1.25 (1.01)	1.19 (1.04)	0.123
Component 6: Use of Sleep Medication	0.00 (0.00)	0.00 (0.00)	0.00 (0.00)	–
Component 7: Daytime Dysfunction	1.09 (1.01)	1.08 (1.01)	1.09 (1.03)	0.516
Global PSQI score	6.85 (2.98)	7.12 (1.76)	6.78 (2.02)	0.013 ^*^
Global PSQI > 5 (n%)	285 (36.9)	155 (41.0)	130 (33.0)	0.021 ^*^
**Clustering of PA, ST and Sleep**	**n (%)**	**n (%)**	**n (%)**	
MVPA < 60 min/d + ST > 120 min/d	534 (69.2)	256 (67.7)	278 (70.6)	0.383
MVPA < 60 min/d + PSQI > 5	192 (24.9)	102 (27.0)	90 (22.8)	0.177
ST > 120 min/d + PSQI > 5	239 (31.0)	128 (33.9)	111 (28.2)	0.087
MVPA < 60 min/d + ST > 120 min/d + PSQI > 5	145 (18.8)	66 (17.5)	79 (20.1)	0.356

*SD* Standard Deviation, ^*^ *p* ≤ 0.05, ^**^ *p* ≤  0.001 ^a^ Frequency responses (0 to 7 days) scored 0–7. Sleep quality assessed using Pittsburg Sleep Quality Index (PSQI)

### Comparison of variables between age categories and type of school attended

Additional Fig. 1 shows the proportion of adolescents engaged in inadequate PA, excess ST, and poor sleep according to the age categories and type of school attended (private vs public schools). Significant differences in PA (χ^2^ = 16.260 *p* < 0.001), ST (χ^2^ = 51.041, p ≤  0.001), sleep duration (χ^2^ = 28.523, p ≤  0.001) and PSQI scores (χ^2^ = 35.779, p ≤  0.001) were observed between younger (10–12 years) and older adolescents (13–15 years). Private school adolescents reported significantly higher ST (χ^2^ = 9.652, *p* = 0.002) and PSQI scores (χ^2^ = 4.4.10, *p* = 0.036).

### Clustering of unhealthy behaviors

More than two-thirds (69.2%) engaged in both PA < 60 min/d and ST > 120 min/d and almost one-third (31.0%) reported having excess ST and poor sleep. The clustering of all three unhealthy behaviors was observed in 18.8% of sampled adolescents. No significant sex differences were observed in the proportion of participants reporting various combinations of unhealthy behaviors (Table 2).

### PA, ST, and sleep as independent risk factors of adiposity

Among girls, the odds of being obesity were significantly higher when PA was < 60 min/d (OR = 1.78, 95% CI 1.54–1.92, *p* ≤ 0.001) and PSQI scores were > 5 (OR = 2.01, 95% CI 1.78–2.25, p ≤0.001) and for central adiposity when ST > 120 min/d (OR = 1.88, 95% CI 1.64–1.98, p ≤ 0.001). Attending private schools, having low PA and increased ST were associated with higher BMI z scores and WHtR in boys. (Table 3).

**Table 3:** Adjusted logistic regression analyses of unhealthy lifestyle behaviors associated with adiposity measures among 10–15 years old adolescents

Independent (Predictor) Variables	Girls (***n*** = 378)		Boys (***n*** = 394)	
	BMI z scores > 1 (***n*** = 136) OR (95% CI)	WHtR > 0.5 (***n*** = 174) OR (95% CI)	BMI z scores > 1 (***n*** = 160) OR (95% CI)	WHtR > 0.5 (***n*** = 152) OR (95% CI)
**Demographic characteristics**				
Age (Ref: 10–12 years)	1.01 (0.92–1.08)	**1.54 (1.31–1.76)** ^**^	**1.11 (1.03–1.22)** ^*^	**0.88 (0.82–0.96)** ^*^
Mother’ education (Ref: < 12th Pass)	**0.88 (0.79–0.95)** ^*^	1**.24 (1.13–1.36)** ^*^	0.92 (0.88–1.05)	0.95 (0.87–1.02)
Mother’ occupation (Ref: Unemployed)	1.03 (0.99–1.11)	**1.12 (1.03–1.28)** ^*^	1.03 (0.95–1.16)	**1.10 (1.04–1.23)** ^*^
Family income (Ref: < 30,000 INR)	1.22 (0.92–1.30)	1.48 (0.82–2.24)	**1.18 (1.03–1.29)** ^*^	0.99 (0.93–1.08)
Type of School Attended (Ref: Public)	**1.08 (1.02–1.14)** ^*^	**2.02 (1.92–2.13)** ^**^	**1.28 (1.21–1.35)** ^*^	**1.63 (1.04–1.99)** ^*^
**Lifestyle behaviors** ^**a**^				
Moderate to vigorous PA (Ref: > 60 min/d)	**1.78 (1.54–1.92)** ^**^	**1.12 (1.03–1.21)** ^*^	**2.10 (1.88–2.23)** ^**^	**1.48 (1.23–1.81)** ^*^
Screen time (Ref: < 120 min/d) Sleep quality (Ref: Global PSQI < 5)	**1.56 (1.35–1.67)** ^*^ **2.01 (1.78–2.25)** ^**^	**1.88 (1.64–1.98)** ^*^ 1.34 (0.73–1.92)	**1.73 (1.26–2.91)** ^**^ **1.44 (1.25–1.62)** ^*^	**2.08 (1.28–4.19)** ^**^ 1.32 (0.68–2.93)
**Clustering of lifestyle behaviors** ^**a**^ (Ref: Participants who do not meet the cluster inclusion criteria)				
Low PA+ excess ST	**1.92 (1.65–2.02)** ^**^	1.03 (0.96–1.17)	**4.13 (3.76–4.24)** ^**^	**2.33 (2.98–3.22)** ^**^
Low PA+ poor sleep	1.05 (0.99–1.19)	**2.19 (1.89–2.26)** ^**^	**1.44 (1.25–1.62)** ^*^	1.03 (0.96–1.17)
Excess ST+ poor sleep	0.89 (0.82–1.06)	1.11 (0.83–1.54)	**1.32 (1.18–1.43)** ^*^	1.16 (0.93–1.26)
Low PA+ excess ST+ poor sleep	**2.88 (2.56–2.90)** ^**^	**1.81 (1.54–1.98)** ^*^	**1.97 (1.24–2.58)** ^**^	**1.18 (1.08–1.25)** ^*^

*Abbreviations*: *BMI* Body Mass Index, *WHtR* Waist to Height Ratio. *OR* Odds Ratio, *CI* Confidence Interval, *PSQI* Pittsburg Sleep Quality Index. *PA* Physical Activity. ST, Screen Time. Ref, Reference

*p ≤ 0.05, ** *p* ≤  0.001

BMI z scores > 1 indicates overweight/ obesity and WHtR > 0.5 indicates central adiposity

^a^Models were adjusted for age, type of school attended, mother’s highest educational qualification, and family’s monthly income

Low PA – Time spent in physical activities < 60 min/d; Excess ST- Time spent using screens > 2 h/d; Poor Sleep - Global PSQI score > 5

Measures that show significant associations with dependent variables are highlighted

### Association of the combination of PA, ST, and sleep behaviors with adiposity measures

The clustering of low PA and poor sleep quality was associated with a higher risk of obesity in girls and of low PA and excess ST with higher BMI and WHtR in boys. Girls who reported clustering of all three unhealthy behaviors had 2.88 times higher odds of obesity and 1.81 times increased odds of having central adiposity. The odds of obesity were 2.10 times higher in boys reporting low PA and 4.13 times higher in those with low PA+ excess ST.

## Discussion

This is one of the few studies to assess the PA, ST, and sleep patterns of adolescents together in a single study and the first to investigate the independent and combined influences of these behaviors on adiposity measures, characterized by increased BMI and WHtR in 10–15 years old adolescents in India. Several key findings related to the prevalence and magnitude of unhealthy lifestyle behaviors and their associations with adolescent obesity emerged from this study. First, our results highlighted the predicament of lower than recommended MVPA (62.1% reported < 60 min/d of PA), a higher amount of time spent on screens (mean ST was 172.4 min/ d), and a clustering of low PA and excess ST in more than two-thirds (69.2%) of the sampled adolescents. Second, the prevalence of sleep insufficiency (sleep duration < 7 h/d) and poor sleep quality (PSQI scores > 5) were observed to be substantially high with a preponderance in girls and older adolescents. Third, sex-stratified regression analyses showed that the predictors of higher BMI and greater central obesity within PA, ST, and sleep patterns vary between adolescent boys and girls. Finally, the findings indicated a co-existence of multiple unhealthy lifestyle risk factors of obesity in the sample and that the clustering of these behaviors significantly increased the likelihood of general and central adiposity in both adolescent boys and girls.

We observed that the odds of obesity were 2.10 times higher when MVPA < 60 min/d, and 1.73 times higher when ST > 2 h/d, but increased to 4.13 times in those who reported a combination of low PA and excess ST. A significantly higher prevalence of obesity in adolescents who spent lesser time in PA and had greater ST has been observed in previous studies [51–53]. The finding that the clustering of low PA and excess ST predicted a higher obesity risk as compared to their independent influences underlines the critical importance of addressing physical inactivity and sedentary behaviors concurrently to avert the immediate and long-term adverse health consequences of obesity in adolescents. Besides the cumulative negative effects of low PA and excess ST on energy expenditure, the high prevalence of obesity can be explained by unhealthy bingeing and snacking habits that are frequently observed among adolescents while engaging in screen-based sedentary activities [14, 54] and a possible displacement of time spent in PA due to excess ST [53, 55]. Furthermore, physiological mechanisms such as suppression of enzymes responsible for glucose and lipid metabolism, altered appetite-regulating hormones, deconditioning of muscular activity, and accumulation of ectopic fat tissues [56–58] may explain the cumulative influences of physical inactivity and prolonged ST on greater general and abdominal adiposity in the sample*.* This is of particular relevance as physical activity patterns and sedentary behaviors during adolescence are known to track into adulthood.

To encourage adequate PA among adolescents, efforts should be taken to adopt school-level strategies such as structured physical education as a part of the academic curriculum, encouragement of students to participate in organized sports, better provision of sports coaching and availability of sports equipment, and having designated play areas or fields at schools. Parents and peers play key roles in encouraging, supporting, and facilitating physical activity in adolescents [59]. In addition to being role models, and providing social support and encouragement to adolescents to engage in organized and unorganized physical activity, other parental factors such as parents’ positive attitude towards benefits of physical activity, active participation in sports, greater family time spent outdoors, and provision of better access and opportunities (example transportation and, training) can help increase the frequency and time spent in physical activities among adolescents [60, 61]. Moreover, the activity patterns of children and their parents are correlated [60, 62], so if the parents are inactive or engage in SB, then the child is also more likely to be inactive. Research indicates that active children are more likely to grow into active adults [52, 63] and that active parents are more inclined to encourage children to participate in recreational and sports activities [64]. This provides opportunities to develop interventions that target adolescent-parent pairs for improvement in adolescent physical activity patterns and obesity management. Family-based interventions that promote activity log keeping or setting family activity goals may add further leverage to school-based PA promotion and ST reduction programs. Also, longitudinal studies to understand how parents’ activity patterns during their childhood can affect their children’s activity levels later merit consideration.

Besides PA and ST, the sleep correlates of adiposity in adolescents were evaluated. A greater prevalence of poor sleep quality in older adolescents (13–15 years old) as compared to the younger participants, ages 10–12 years in this study can be attributed to alterations in school timings, increased academic demands, and greater electronic device usage [65, 66] combined with a known preference for eveningness over morningness [67] and an advancing pubertal status that is associated with changes in circadian timing and melatonin secretion [68]. Sleep deprivation may predispose to obesity by altering the activity of appetite-controlling hormones - leptin and ghrelin and by elevating the cortisol levels, both of which have been linked to an increase in energy intake and reduction in energy expenditure [69, 70]. In our study, having PSQI scores> 5 was significantly associated with higher odds of obesity, highlighting a need for regular screening of sleep disturbances and consistent education efforts to foster improved bedtime hygiene and better sleep quality in adolescents in India. 

In this study, the sex differences in PA, ST, and sleep and the influences of these behaviors on adiposity measures were explored. Girls reported higher ST, decreased sleep duration, and greater PSQI scores as compared to boys. Given that excess ST and insufficient sleep are independently associated with an increased risk of developing non-communicable diseases including obesity, the combination of these unhealthy behaviors might predispose adolescent girls to a higher risk of poorer health outcomes. We used sex-stratified regression analyses to determine factors associated with BMI and WHtR. The majority of previous studies had adjusted the regression models for sex [5, 71]. However, this may undermine the pervasive gender differences in prevalence and factors associated with obesity, and limit the scope of obesity prevention interventions in adolescents. In this study, the clustering of low MVPA with poor sleep predicted greater central adiposity in girls, and the clustering of low MVPA with excess ST was observed to be associated with a higher BMI and greater WHtR in boys. The combination of low PA, excess ST, and poor sleep increased the odds of bother generalized and central obesity in girls but only an increased BMI in boys. These results warrant further investigation into factors that might contribute to the gender differences in PA, sedentary and sleep behaviors of adolescents so that appropriate policy and practice recommendations can be developed.

The novelty of this study lies in evaluating the combined effect of unhealthy behaviors on adiposity measures in Indian adolescents. Previous studies that were conducted in high-income countries have reported favorable associations between meeting movement behavior recommendations and better health outcomes in adolescents [2, 9, 72]. Yet, the independent and combined influences of non-adherence to MVPA, ST, and sleep guidelines on body weight status and central obesity indicators in adolescents remain limited. The finding of our study that clustering of unhealthy lifestyle behaviors exerts pertinent influences on the risk of obesity attains relevance for adolescents residing in the low middle-income countries such as India where the impact of urbanization and economic growth on lifestyles, particularly in PA, screen time and SB is still underway [17, 73] and little is known about the influence of engaging in a specific lifestyle behavior on other behaviors.

As such, the multifactorial nature of obesity accentuates the importance of identifying and addressing the risk factors that contribute to obesity in adolescents. The results of this study reiterate the critical importance of targeting each component of the ‘movement continuum’ [5] that occurs from low movement activities such as sleep to activities that involve higher movements or energy expenditures such as moderate to vigorous physical activities whilst addressing the interplay between these behaviors to facilitate greater improvements in obesity-related risk behaviors among adolescents in India. Though a majority of previous evidence suggests that unhealthy lifestyle behaviors typically precede obesity and metabolic disorders in adolescents, there is emerging research to indicate that obesity may trigger a self-perpetuating vicious circle of low habitual physical activity, excess time spent in sedentary behaviors, poor sleep quality, and increasing adiposity, mediated through genetic, environmental and psychosocial factors [33, 34, 38]. Examining the bi-directional associations of obesity with a decline in physical activity and increase in screen time and sleep disturbances may present expedient targets for future interventions among adolescents in India as elsewhere.

A few limitations of our study must also be mentioned. First, the objective assessments of PA, ST, or sleep using accelerometers could not be done due to resource constraints. Second, though we used validated tools to estimate the activity and sleep patterns, the data was self-reported and can be subject to recall and social desirability bias. Third, only maternal factors such as education and occupation were assessed as indicators of familial support for engaging in healthy lifestyle behaviors. Finally, the convenience sampling method and the cross-sectional design of the study may limit the interpretations of associations between variables and formulation of hypotheses for future experimental studies.

However, the strength of this study is that it is the first large-scale survey to evaluate the combined effects of low physical activity levels, excess sedentary behaviors, and poor sleep quality on body weight status and central adiposity indicators in adolescents in India, albeit in a non-representative manner. Adolescents in the early and mid-adolescence and attending both private and public schools were selected and the sex, age, and socioeconomic differences in the activity, sedentary, and sleep behaviors were assessed. Also, we used BMI as well as WHtR to determine obesity, providing evidence of demographic and lifestyle-related risk factors associated with general and central adiposity in adolescents in India. Practical applications of this study include regular assessments of simple anthropometric measurements to characterize obesity in adolescents in school settings, early identification of sleep disturbances and excess sedentary behaviors using validated questionnaires, and designing educational interventions and feasible activity guidelines to foster health-promoting lifestyle habits and reduce risk of immediate and long-term consequences of adiposity in the pediatric population.

## Conclusion

Adolescence is a critical phase of life when hormonal, physiological, and lifestyle factors contribute to alterations in metabolic pathways to support increased nutrient requirements and facilitate growth spurt. In adolescents with obesity, these manifestations may continue to persist and eventually contribute to an early onset of metabolic derangements such as hyperinsulinemia, hypertension, and other cardiometabolic disorders [31, 72]. The results of our study provide valuable evidence that the modifiable lifestyle factors of adolescent obesity such as reduced PA levels, excess time spent in screen-based SB, and poor sleep quality tend to co-occur and can further aggravate the risk of obesity as compared to the independent effect of these behaviors. Research thus far has mainly focused on programs that promote healthy PA, sedentary, and sleep behaviors separately to address the burgeoning challenge of obesity prevention in adolescents in India. Future prevention efforts must consider a better understanding of the complex interactions that ensue between these unhealthy behaviors and develop broader and integrated interventions that leverage the cumulative benefits of being active, less sedentary, and getting enough sleep to moderate their individual influences on adiposity measures in adolescents.

## Supplementary Information

**Additional file 1.**

**Additional file 2.**

## Data Availability

The research data included in the analysis are the authors’ content. The datasets used and/or analyzed during the present study are available from the corresponding author on reasonable request.
