# Supplementary material for: Independent and combined influences of physical activity, screen time, and sleep quality on adiposity indicators in Indian adolescents
Source: BMC Public Health. 2021 Nov 15;21:2093. doi: 10.1186/s12889-021-12183-9 (PMC8591930; doi:10.1186/s12889-021-12183-9)
Supplement: Supplementary file 1 — Additional file 1. [file 12889_2021_12183_MOESM1_ESM.docx]

**Additional Table 1: Sleep quality of 10-15 years old adolescents, as measured using Pittsburg Sleep Quality Index (PSQI)**

| **Sleep duration and quality components** | **Overall (n=772)** | **Girls (n=378)** | **Boys (n=394)** | **p value** |
| --- | --- | --- | --- | --- |
| **Average time taken to fall asleep (minutes/d)**  **Average duration of actual sleep (hours/d)**  **Subjective sleep quality, n (%)**  Very good  Fairly good  Fairly bad  Very bad | 24.33 (12.80)  7.21 (1.14)  482 (62.4)  142 (18.4)  99 (12.8)  49 (6.3) | 23.59 (10.12)  7.01 (1.09)  232 (61.4)  78 (20.6)  42 (11.1)  26 (6.9) | 24.80 (11.71)  7.29 (1.10)  250 (63.5)  64 (16.2)  57 (14.5)  23 (5.8) | 0.125  0.002 ^*^  0.547 |
| **Sleep latency (min), n (%)**  <15  15-30  31-60  >60  **Sleep duration (h), n (%)**  >7  6-7  5-6  <5 | 404 (52.3)  218(28.2)  86 (11.1)  64 (8.3)  411 (53.2)  188 (24.4)  121 (15.7)  52 (6.7) | 212 (56.1)  103(27.2)  48 (12.7)  15 (4.0)  198 (52.4)  83 (22.0)  76 (20.1)  21 (5.6) | 192 (48.7)  115 (29.2)  38 (9.6)  49 (12.4)    213 (54.1)  105 (26.6)  45 (11.4)  31 (7.9) | 0.126  0.012^*^ |
| **Habitual sleep efficiency, n (%)**  >85  75-84  65-74  <65 | 505 (65.4)  208 (26.9)  41 (5.3)  18 (2.3) | 253 (66.9)  98 (25.9)  19 (5.0)  8 (2.1) | 252 (64.0)  110 (27.9)  22 (5.6)  10 (2.5) | 0.478 |
| **Sleep disturbances (sum of 9 items),** n (%)  0  1-9  10-18  19-27 | 195 (25.3)  512 (66.3)  58 (7.5)  7 (0.9) | 90 (23.8)  249 (65.9)  34 (9.0)  5 (1.3) | 105 (26.6)  263 (66.8)  24 (6.1)  2 (0.5) | 0.063 |
| **Daytime dysfunction, n (%)**  **Trouble staying awake while driving, eating meals, or engaging in social activity**  Not during past month  Less than once a week  Once or twice a week  Three or more times a week  **Problem to keep up enough enthusiasm to get things done, n (%)**  No problem at all  Only a very slight problem  Somewhat of a problem  A very big problem | 649 (84.1)  77 (10.0)  41 (5.3)  5 (0.6)  502 (65.0)  222 (28.8)  45 (5.8)  3 (0.4) | 303 (80.2)  49 (13.0)  22 (5.8)  4 (1.1)  248 (65.6)  108 (28.6)  21 (5.6)  1 (0.3) | 346 (88.3)  28 (7.1)  19 (4.8)  1 (0.3)  257 (65.2)  114 (28.9)  24 (6.1)  2 (0.5) | <0.001 ^**^  0.815 |
| **Global PSQI, n (%)**  ≤ 5  > 5 | 487 (63.1)  285 (36.9) | 223 (59.0)  155 (41.0) | 264 (67.0)  130 (33.0) | 0.021^*^ |

PSQI, Pittsburg Sleep Quality Index. ^*^ p ≤ 0.05, ^**^ p < 0.001
