# Supplementary material for: Independent and combined influences of physical activity, screen time, and sleep quality on adiposity indicators in Indian adolescents
Source: BMC Public Health. 2021 Nov 15;21:2093. doi: 10.1186/s12889-021-12183-9 (PMC8591930; doi:10.1186/s12889-021-12183-9)
Supplement: Supplementary file 2 — Additional file 2. [file 12889_2021_12183_MOESM2_ESM.docx]

**Additional Figure 1**: Physical activity levels, sedentary behaviors and sleep quality of adolescents according to age and type of school attended
